# Impending paradoxical embolism after type A aortic dissection surgery: mechanistic insight into the role of pericardial effusion and aortic geometry — a case report

**DOI:** 10.1093/jscr/rjag680

**Published:** 2026-08-08

**Authors:** Takateru Yamamoto, Haruki Tanaka, Megumi Fuke, Tatsuichiro Seto

**Affiliations:** Division of Cardiovascular Surgery, Department of Surgery, Shinshu University School of Medicine, Matsumoto, Nagano 390-8621, Japan; Division of Cardiovascular Surgery, Department of Surgery, Shinshu University School of Medicine, Matsumoto, Nagano 390-8621, Japan; Division of Cardiovascular Surgery, Department of Surgery, Shinshu University School of Medicine, Matsumoto, Nagano 390-8621, Japan; Division of Cardiovascular Surgery, Department of Surgery, Shinshu University School of Medicine, Matsumoto, Nagano 390-8621, Japan

**Keywords:** acute pulmonary embolism, impending paradoxical embolism, patent foramen ovale, type A acute aortic dissection

## Abstract

A 72-year-old woman with type A acute aortic dissection and a patent false lumen underwent emergency hemiarch replacement. On postoperative Day 13, routine contrast-enhanced computed tomography revealed an impending paradoxical embolism (IPE) and a right main pulmonary embolism. Surgical pulmonary thrombectomy and patent foramen ovale closure were performed. This case provides mechanistic insight into IPE development in the postoperative period. Early-phase hypercoagulability after acute aortic dissection, increased right atrial pressure due to pulmonary embolism, and anatomical factors such as pericardial effusion and altered aortic geometry after graft replacement may have facilitated right-to-left shunting through a previously undetected or functionally latent patent foramen ovale. The patient was discharged on postoperative Day 14 (hospitalization Day 27) and remained asymptomatic without recurrence during 5-year follow-up. This case highlights the importance of considering IPE after type A acute aortic dissection repair and supports routine postoperative imaging to detect clinically silent but life-threatening complications.

## Introduction

Acute pulmonary embolism (PE) in the presence of a patent foramen ovale (PFO) is a risk factor for paradoxical systemic embolism. An impending paradoxical embolism (IPE), defined as a thrombus straddling a PFO, is a rare but life-threatening condition requiring urgent intervention [1]. We report a rare case of IPE detected after hemiarch replacement for type A acute aortic dissection (TAAD) successfully treated with surgical thrombectomy and PFO closure.

## Case report

**Figure 1:**
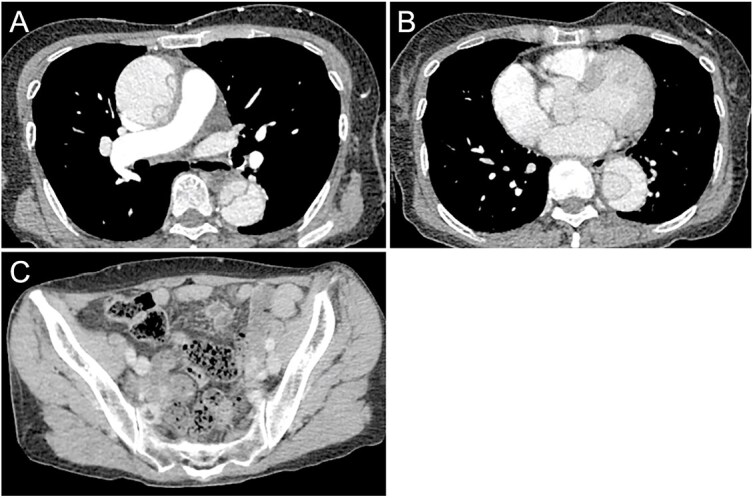
Contrast-enhanced CT showing type A acute aortic dissection. No thrombus was observed in the iliac veins.

A 72-year-old woman presented with chest pain and dizziness. On admission, her vital signs were stable, and the Glasgow Coma Scale score was E4V5M6. Laboratory testing revealed leukocytosis (17 040/μl) and a markedly elevated D-dimer level (58.7 μg/ml). Contrast-enhanced computed tomography (CT) revealed a TAAD extending from the ascending aorta to the descending thoracic aorta with a patent false lumen and ascending aortic dilatation to 45 mm. No significant branch vessel malperfusion was observed (Fig. 1).

Emergency hemiarch replacement was performed. Intraoperative transesophageal echocardiography (TEE) showed no evidence of a PFO. The postoperative course was initially uneventful.

**Figure 2:**
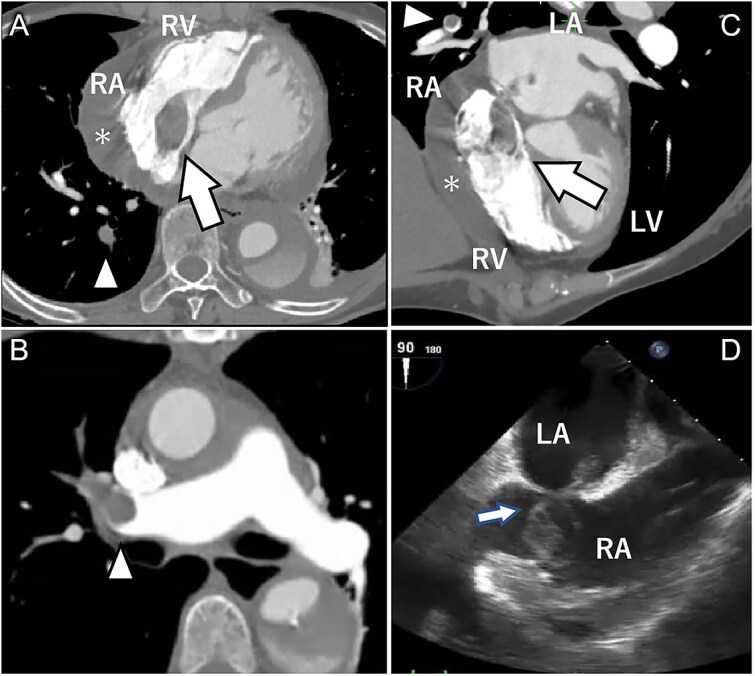
Contrast-enhanced CT and intraoperative transesophageal echocardiogram demonstrating an impending paradoxical embolism (arrow), a pulmonary embolism in the right main pulmonary artery (▲), and pericardial effusion (^*^). LA, left atrium; RA, right atrium; LV, left ventricle; RV, right ventricle.

On postoperative day (POD) 13, routine follow-up contrast-enhanced CT performed before discharge confirmed successful aortic anastomosis; however, it revealed a thrombus straddling a PFO and PE involving the right main and upper pulmonary arteries (Fig. 2). No systemic embolism was detected. The patient had mild lower-extremity edema. Despite standard postoperative thromboprophylaxis, the D-dimer level increased to 17.3 μg/ml, leading to a diagnosis of IPE.

Surgical pulmonary thrombectomy with PFO closure was performed on POD 14. Cardiopulmonary bypass was established using bicaval venous drainage and arterial perfusion through the ascending aortic graft. Although TEE demonstrated a thrombus straddling the PFO before cardiac arrest, no intracardiac thrombus was identified after right atriotomy. The PFO measured approximately 10 mm. The left atrium and ventricle were carefully inspected via a transseptal approach, revealing no thrombus. A large thrombus was instead identified in the main pulmonary artery, and residual thrombi were removed from both pulmonary arteries. The atrial septum was closed directly.

Postoperatively, anticoagulation therapy with heparin was initiated, later, the patient was transitioned to a direct oral anticoagulant. Follow-up CT confirmed resolution of PE. The patient was discharged on POD 14 (hospitalization Day 27) and remained asymptomatic without recurrence during a 5-year follow-up.

## Discussion

Paradoxical embolism occurs when venous thrombi enter the systemic circulation through a right-to-left shunt, most commonly via a PFO. To date, only one case of IPE following cardiac surgery has been reported, highlighting the rarity of this condition in the postoperative setting [2].

In the present case, IPE was identified in the early postoperative period following TAAD surgery. Intraoperative TEE did not detect a PFO; however, the absence of a detectable shunt does not exclude its presence, as interatrial shunting can be dynamic and dependent on transient changes in right atrial pressure [3]. Clinically significant right-to-left shunting may become apparent under postoperative hemodynamic stress in cases with a previously undetected or functional silent PFO, particularly in conditions such as cardiac tamponade, positive-pressure ventilation, or right ventricular dysfunction [4, 5].

The development of IPE in this case was likely triggered by a combination of early-phase hypercoagulability following acute aortic dissection, increased right atrial pressure, and anatomical factors. First, acute aortic dissection promotes a prothrombotic state through thrombin formation within the false lumen, thereby facilitating thrombus formation [6]. Despite standard thromboprophylaxis, extensive thromboembolism developed, suggesting the significant role of dissection-related hypercoagulability.

**Figure 3:**
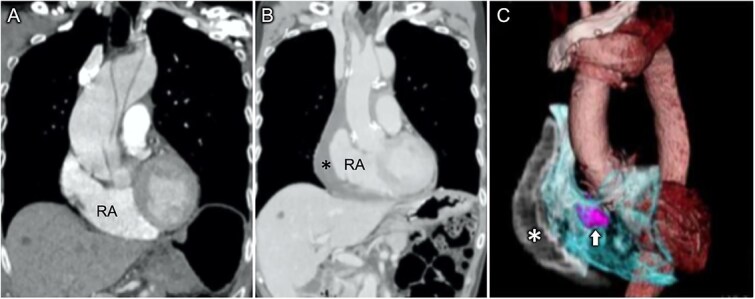
Contrast-enhanced CT showing the anatomical relationship between the ascending aorta and the right atrium. Postoperative dilatation and elongation of the ascending aorta, along with pericardial effusion, may contribute to right atrial compression and facilitate right-to-left shunting. Arrow, impending paradoxical embolism; ^*^, pericardial effusion; RA, right atrium.

Second, postoperative anatomical and hemodynamic changes also may have contributed. Elevation of right atrial pressure is a key mechanism underlying paradoxical embolism. Structural factors, such as postoperative dilatation and elongation of the ascending aorta after graft replacement, may facilitate right-to-left shunting (Fig. 3) [7].

Platypnea-orthodeoxia syndrome represents a related mechanism in which anatomical distortion facilitates shunt flow [8]. Postoperative pericardial effusion, which was visible on CT may have contributed to elevated right atrial pressure and facilitated right-to-left shunting.

Surgical thrombectomy with PFO closure was selected because thrombolysis was contraindicated due to recent major surgery [9]. In the present case, intraoperative migration of the thrombus into the pulmonary circulation cannot be excluded; therefore, careful venous drainage and minimal manipulation of the right atrium may help reduce the risk of thrombus migration.

This case provides important clinical implications. TAAD and its surgical repair create a prothrombotic and hemodynamically complex environment, predisposing patients to IPE. When routine postoperative contrast-enhanced CT reveals pericardial effusion or ascending aorta dilatation, clinicians should be aware of the potential for subsequent IPE.

In conclusion, we report a rare case of IPE occurring 13 days after hemiarch replacement for TAAD. Surgical thrombectomy and PFO closure were successfully performed. Hypercoagulability following acute aortic dissection, in combination with postoperative haemodynamic and anatomical factors, may contribute to the development of this condition. Clinicians should be aware of this potential complication, particularly in the presence of pericardial effusion and altered aortic geometry.

## Data Availability

No datasets were generated or analysed during the current study.
